# Inclusive Clinical Trials in Alzheimer’s Disease: A Latin American Perspective from LatAm FINGERS

**DOI:** 10.1002/alz.085948

**Published:** 2025-01-09

**Authors:** Lucia Crivelli, Ismael Luis Calandri, Paulo Caramelli, Francisco Lopera, Ricardo Nitrini, Ana Luisa Sosa, Rosa Maria Salinas‐Contreras, Claudia Kimie Suemoto, Lina M Velilla, Monica Sanches Yassuda, Gustavo Sevlever, Daisy M Acosta, Ana Charamelo, María Isabel Cusicanqui, Nilton Custodio, Carolina Delgado, Lissette Duque, Ivonne Z. Jimenez Velazquez, Jorge M Leon‐Salas, Heather M Snyder, Miia Kivipelto, Maria C. Carrillo, Ricardo Allegri, LatAm‐FINGERS Consortium

**Affiliations:** ^1^ Fleni, Buenos Aires, Buenos Aires Argentina; ^2^ Fleni, Buenos Aires Argentina; ^3^ Behavioral and Cognitive Research Group, Faculdade de Medicina, Universidade Federal de Minas Gerais, Belo Horizonte Brazil; ^4^ Neurosciences Group of Antioquia, University of Antioquia, Medellin Colombia; ^5^ Faculdade de Medicina da Universidade de São Paulo, São Paulo Brazil; ^6^ Dementias Laboratory, National Institute of Neurology and Neurosurgery, Mexico City, DF Mexico; ^7^ Division of Geriatrics, Department of Internal Medicine, University of Sao Paulo Medical School, São Paulo, São Paulo Brazil; ^8^ Neurosciences Group of Antioquia, University of Antioquia, Medellín Colombia; ^9^ University of São Paulo, São Paulo Brazil; ^10^ Universidad Nacional Pedro Henriquez Ureña, Santo Domingo, Distrito Nacional Dominican Republic; ^11^ Departamento de Neuropsicología, Facultad de Medicina‐Hospital de Clínicas, Universidad de la República, Montevideo Uruguay; ^12^ Centro Neurológico Mente Activa, La Paz Bolivia (Plurinational State of); ^13^ Instituto Peruano de Neurociencias, Lima, Lima Peru; ^14^ Universidad de Chile, Santiago, Region Metropolitana Chile; ^15^ Neuromedicenter, Quito Ecuador; ^16^ University of Puerto Rico, School of Medicine, San Juan, Puerto Rico, PR USA; ^17^ Clínica Bíblica Hospital, San José Costa Rica; ^18^ Alzheimer’s Association, Chicago, IL USA; ^19^ Kuopio University Hospital, Kuopio Finland; ^20^ Karolinska Institutet, Stockholm Sweden; ^21^ Imperial College London, London United Kingdom; ^22^ FLENI, Buenos Aires Argentina

## Abstract

**Background:**

The LatAm‐FINGERS trial marks a pioneering initiative as the first non‐pharmacological clinical trial encompassing participants from 12 Latin American countries, including Argentina, Brazil, Bolivia, Chile, Colombia, Costa Rica, Ecuador, Dominican Republic, Mexico, Peru, Puerto Rico, and Uruguay. This initiative represents a significant advancement in promoting inclusivity and diversity in clinical trial recruitment, particularly in underserved populations.

**Method:**

The LatAm‐FINGERS trial is a multicenter randomized clinical trial evaluating a lifestyle intervention tailored for the Latin American population. Innovative and culturally sensitive recruitment methods were employed, utilizing social media, community outreach, and partnerships with religious groups to ensure inclusivity. The multidomain intervention includes diet, physical exercise, cognitive training, and health coaching and socialization. The intervention was delivered in teams of ten, with adherence strategies including moderated chat groups, dietary adaptations to local customs, shared cultural celebrations, recognition awards, personalized video tutorials, engagement calls, and motivational elements. These approaches facilitated participant retention and engagement despite socio‐economic challenges.

**Result:**

Despite challenges such as transportation difficulties and risks of violence in certain regions, the trial effectively implemented prevention strategies across a diverse demographic spectrum. Recruitment for LatAm‐FINGERS began in December 2021. To date, 1186 participants (98.8%) have been randomized into the intervention group. The average age of participants is 69 (SD = 2.5) years, with an average educational level of 12.4 (SD = 3.4) years, and an MMSE score of 27.32 (SD = 1.68). The diversity of the Latin American population is evident in the self‐reported ethnicity data: 57.1% Mestizo, 27.7% White, 5.5% Mixed, 2% Black, 1% Native American, and 5.7% other.

**Conclusion:**

Preliminary findings from LatAm‐FINGERS highlight the feasibility of conducting intervention trials within diverse populations. These findings underscore the importance of creating recruitment strategies that effectively engage underrepresented groups. This contributes significantly to global Alzheimer’s disease research efforts. The trial’s experience offers a valuable model for inclusive enrollment practices in Alzheimer’s disease and related dementia research, aligning with contemporary trends toward equitable and evidence‐based trial design.

